# Effect of Modified Triple-Layer Application on the Bond Strength of Different Dental Adhesive Systems to Dentin

**DOI:** 10.3390/jfb14100522

**Published:** 2023-10-17

**Authors:** Rim Bourgi, Naji Kharouf, Carlos Enrique Cuevas-Suárez, Monika Lukomska-Szymańska, Walter Devoto, Cynthia Kassis, Omar Hasbini, Davide Mancino, Youssef Haikel, Louis Hardan

**Affiliations:** 1 Department of Restorative Dentistry, School of Dentistry, Saint-Joseph University, Beirut 1107 2180, Lebanon; cynthia.kassis@usj.edu.lb (C.K.); louis.hardan@usj.edu.lb (L.H.); 2 Department of Biomaterials and Bioengineering, INSERM UMR_S 1121, University of Strasbourg, 67000 Strasbourg, France; mancino@unistra.fr (D.M.); youssef.haikel@unistra.fr (Y.H.); 3 Department of Endodontics and Conservative Dentistry, Faculty of Dental Medicine, University of Strasbourg, 67000 Strasbourg, France; 4 Dental Materials Laboratory, Academic Area of Dentistry, Autonomous University of Hidalgo State, San Agustín Tlaxiaca 42160, Mexico; cecuevas@uaeh.edu.mx; 5 Department of General Dentistry, Medical University of Lodz, 92-213 Lodz, Poland; monika.lukomska-szymanska@umed.lodz.pl; 6 Independent Researcher, 16030 Sestri Levante, Italy; walter@walterdevoto.com; 7 Department of Prosthetic Dentistry, School of Dentistry, Saint-Joseph University, Beirut 1107 2180, Lebanon; omar.hasbini@net.usj.edu.lb; 8 Pôle de Médecine et Chirurgie Bucco-Dentaire, Hôpital Civil, Hôpitaux Universitaire de Strasbourg, 67000 Strasbourg, France

**Keywords:** active application, aging, bond strength, etch-and-rinse adhesives, self-etch adhesives, universal adhesives

## Abstract

The goal of this article was to assess the effect of modified triple-layer application (MTLA) in conjunction with the active bonding technique on the bond strength of four adhesive systems to dentinal substrate. The adhesives tested were Prime&Bond Universal (PBU), OptiBond Universal (OBU), OptiBond FL (OBFL), and Clearfil SE (CSE). The adhesives were applied according to the following strategies: single active application (A) and triple adhesive layer application including Active–Passive–Passive (APP); AAP; and AAA. The micro-tensile bond strength test was evaluated following 24 h or 6 months of storage. The composite–dentin interface morphology was investigated using scanning electron microscopy. The data were statistically analyzed with a significance level of α = 0.05. At 24 h of aging, all of the factors tested were not significant (*p* > 0.05) for CSE. For OBFL, OBU, and PBU, statistically higher values were observed for the A technique (*p* < 0.05). Plus, there were no significant variances between the APP, AAP, and AAA techniques (*p* > 0.05) for OBFL and PBU. However, for OBU, there were no significant differences between the A and AAA techniques (*p* > 0.05). After 6 months of aging, the A technique showed statistically higher values when compared to the other techniques (*p* < 0.01), except for OBFL, where the A and AAA techniques showed promising outcomes. When comparing the bond strength values of 24 h and 6 months, only for PBU, all of the techniques used resulted in bond strength stability over time (*p* > 0.05). Thicker adhesive layers were observed when MTLA was applied. Only the OBFL adhesive showed the formation of resin tags in all of the modalities tested. The bonding performances of the different application techniques used were material-dependent.

## 1. Introduction

The intrinsic wet nature of dentin makes it a challenging substrate for resin composite bonding [1]. Moreover, most of the time in clinical settings, the dentin is still covered with smear layers, which prevent adhesive molecules from penetrating the dentin [2]. Consequently, removing or altering this layer by means of an acid etching stage preceding the application of the bonding agent is crucial for the formation of the hybrid layer (HL) [3]. Dental adhesives’ ability to bond to dentin relies on the HL creation to warrant a strong bond between the resin monomers and dentin structure [4]. Lately, an additional alteration of these adhesive systems, the so-called universal adhesives (UAs), has been familiarized into the dental market. Unlike their forerunners, these adhesives can be used in both self-etch (SE) and etch-and-rinse (E&R) strategies [3,4,5].

Further improvements to dental adhesives involved initiating new molecules into the components of adhesive systems: 10-methacryloyloxydecyl dihydrogen phosphate (10-MDP), glycero-phosphate dimethacrylate (GPDM), 4-methacryloxyethyl trimellitic acid (4-MET), and N-Phenyl-p-phenylenediamine (phenyl-P). They were designated as functional monomers and were considered to chemically adhere to calcium in hydroxyapatite (HAp) [5]. Amongst these molecules, the 10-MDP monomer was stated to acquire the toughest chemical adhesion possible to HAp, creating constant and hydrolysis-resistant calcium salts because of the nano-layering process [6]. Therefore, an extra layer of adhesive application must be considered as a fundamental step in dental clinics. Furthermore, 10-MDP necessitates a suitable time of 20 s for its chemical interaction to take place; yet, employing a second layer of such a functional monomer without curing the first coat warrants the first layer to sufficiently interact with HAp and consequently encourages further bonding [7]. A double-layer application might be explained by the following theory: the first coat of the adhesive system demineralizes the dentinal substrate via etching using phosphoric acid or acidic monomers and thus could be rapidly buffered by the Hap [2]; the additional layers of non-photopolymerized acidic monomers might successively increase etching by rising the concentration of acid components. The extra infiltration of resin monomers might concurrently appear due to the added amount of adhesive system [8].

The adhesives are usually able to create an HL with both immediate and medium-term high bond strength. However, losses of dentin-bonded interface integrity and bond strength are usually seen after aging [9]. There are many factors involved in this loss of bonding efficacy, including an unsatisfactory resin infiltration of the dentinal structure, phase separation, as well as a low level of adhesive polymerization, all of which might lessen the durability of a bonded interface [4]. All of these variables could be avoided by creating a gold pattern application protocol designed to maximize the effectiveness of contemporary adhesive systems. Currently, there are not any standard adhesive application protocols to enhance the longevity of resin–dentin bonds created by adhesive systems.

Nevertheless, the hybridized dentin does not fully cover the depth of the demineralized dentin. The true HL is actually thinner than it appears in the scanning electron microscopy (SEM), since a demineralized dentin zone still exists, especially at the bottom [10]. However, this thinner HL and resulting inadequate stress-breaking action might be recompensed using a thicker adhesive coat at the top [11]. Former analyses with diverse adhesive systems stated that promising outcomes with the active application (AA) modality or an additional coat of the adhesive layer could be obtained to enhance their bond performance to dentin [12,13]. An active adhesive application can achieve a higher rate of monomer infiltration inside the collagen network, whereas an extra bonding layer might rise the thickness of the adhesive layer, therefore improving the distribution of stress when a load is applied and decreasing the degradation of the HL. The use of multiple-layer application (MLA) increased the immediate bond strength, but this result could not be observed after aging [13]. Thus, it was deemed essential to know how many adhesive layers should be applied to the dentin structure in order to improve the bond performance. Previous papers proposed that double or triple adhesive coats enhance the bond strength by enabling monomer diffusion into the HL and increasing chemical interactions [8,14]. Another variation of the adhesive application can be found in the adhesive technique without polymerization, with favorable results [15,16].

Correspondingly, there are no previously published studies in the literature based on the adhesive layer application including triple adhesive layers in conjunction with the “active bonding technique” (ABT), called modified triple-layer application (MTLA). Appropriately, a description of this novel technique could be interesting for dentists aiming for a better adhesion to dentin. Hence, the aim of this article was to assess the effect of the MTLA of four adhesive systems on the dentin bond strength as well as the correlation between these parameters at 24 h and 6 months of aging. According to the null hypothesis, there is no effect of the ABT with MLA on the bond strength and morphological properties of adhesive systems to dentin substrates.

## 2. Materials and Methods

**Figure 1:**
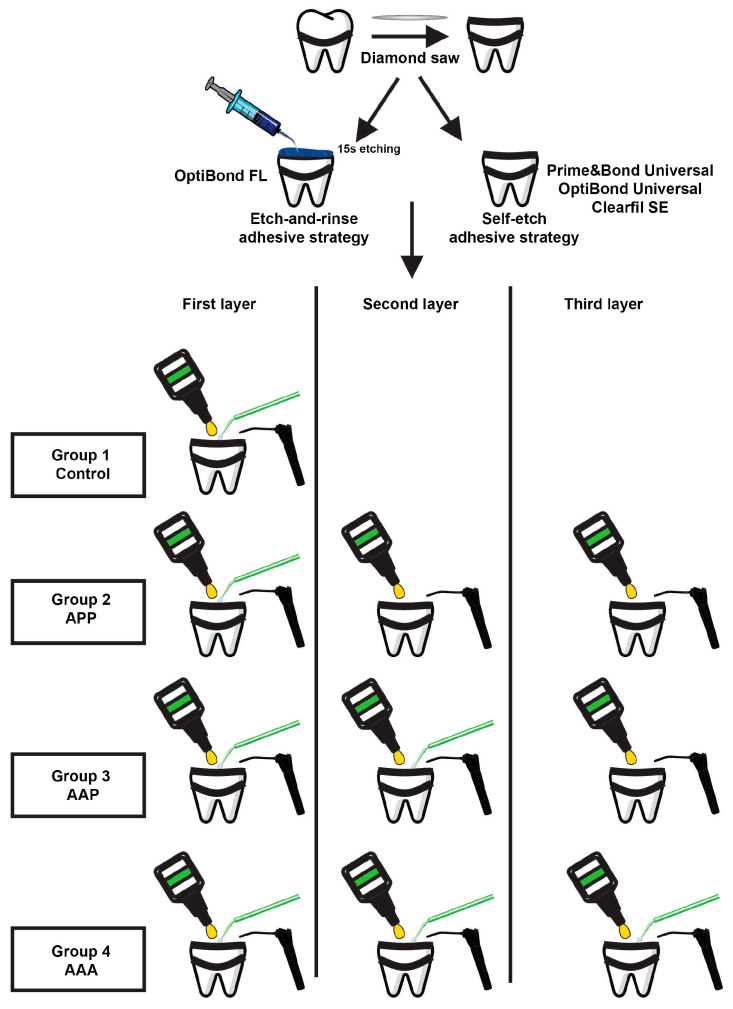
Representation of the four groups tested in this research. Single active application (A); triple application, Active–Passive–Passive (APP); triple application, Active–Active–Passive (AAP); triple application, Active–Active–Active (AAA).

The study protocol was agreed upon by the ethical team of the dental faculty at the Saint-Joseph University of Beirut, Lebanon (FMD-221; reference number: #USJ-2022-140). A representation of the study groups is described in Figure 1.

### 2.1. Tooth Specimen Preparation

Eighty (n = 80) sound human molars without any signs of cracking in the enamel or caries were extracted for orthodontic reasons and examined for micro-tensile bond strength (μTBS). These molars were collected, freed of soft tissue, and kept at 4 °C for a month in a solution of 0.2% sodium azide to prevent microbial growth. Then, roots were segmented, and their crowns were fixed in gypsum to reveal the buccal enamel. Later, enamel substrate was abraded using an orthodontic grinder (Essencedental, Araraquara, SP, Brazil) until exposing and covering a flat medium dentin surface that conserved 4 mm of dentin in thickness. A regular and standardized smear layer was produced on the dentin by means of P320 silicon carbide sandpaper (SiC) during 1 min, under water irrigation, with a grinder–polisher (Buehler Ltd., Lake Bluff, IL, USA) at a motor speed of 70 rpm.

### 2.2. Bonding Procedure

Following dentinal surface exposure, four groups, based on the adhesive layer application including the triple adhesive layers and ABT, were randomly formed. One coat (control) and three coats of adhesives were applied without photopolymerization after each application. ABT of adhesives to dentinal substrate was performed for 20 s manually by a single operator using a microbrush applicator (Kerr, Orange, CA, USA). Additionally, the magnitude of force to be applied during rubbing action was standardized by one investigator who performed the adhesion process.

Two UAs (Prime&Bond Universal, PBU, Dentsply DeTrey GmbH, Konstanz, Germany; OptiBond Universal, OBU, Kerr Co, Orange, CA, USA), one three-step E&R adhesive system (OptiBond FL, OBFL, Kerr Co, Orange, CA, USA), and one two-step SE adhesive system (Clearfil SE, CSE, Kuraray Noritake Dental Inc., Tokyo, Japan) were assessed. The compositions of the adhesive systems tested in the current research are shown in Table 1. 

The number of teeth per group (n = 5) was expected based on a preceding analysis [17] that assessed the effect of MLA on the dentinal bond strength in a comparative study design with four independent groups; there was a 5.87 minimum detectable difference in means, a 1.54 standard deviation, a power of 0.8, and α = 0.05. For the first group (control), one coat of all the tested adhesives was applied for 20 s with the ABT (single active application (A)). For the second group, MTLA was applied as follows: application of the first layer with the ABT, application of the second layer without the ABT, and application of the third layer without the ABT (group 2 of triple application: Active–Passive–Passive (APP)). For the third group, MTLA was applied as follows: application of the first layer with the ABT, application of the second layer with the ABT, and application of the third layer without the ABT (group 3 of triple application: Active–Active–Passive (AAP)). Further, for group four, the first, second, and third layers were used with the ABT (group 4 of triple application: Active–Active–Active (AAA)).

Solvent evaporation after each layer of primer or adhesive was performed for 5 s to 10 s by means of an air-drying syringe until there was no visible movement of the material. Next, bonding agents were polymerized for 20 s at room temperature using a Light-Emitting Diode (LED) multiwave light-curing unit, CuringPen-E (Eighteeth, Changzhou, China), calibrated at 1000 mW/cm^2^. After bonding to the flat dentinal surfaces, three increments of resin composites (Reflectys, Itena Clinical, Paris, France) were created with a maximum thickness of 2 mm each. Each coat was photopolymerized for 20 s with the same light-curing unit.

### 2.3. Micro-Tensile Bond Strength Testing

**Figure 2:**
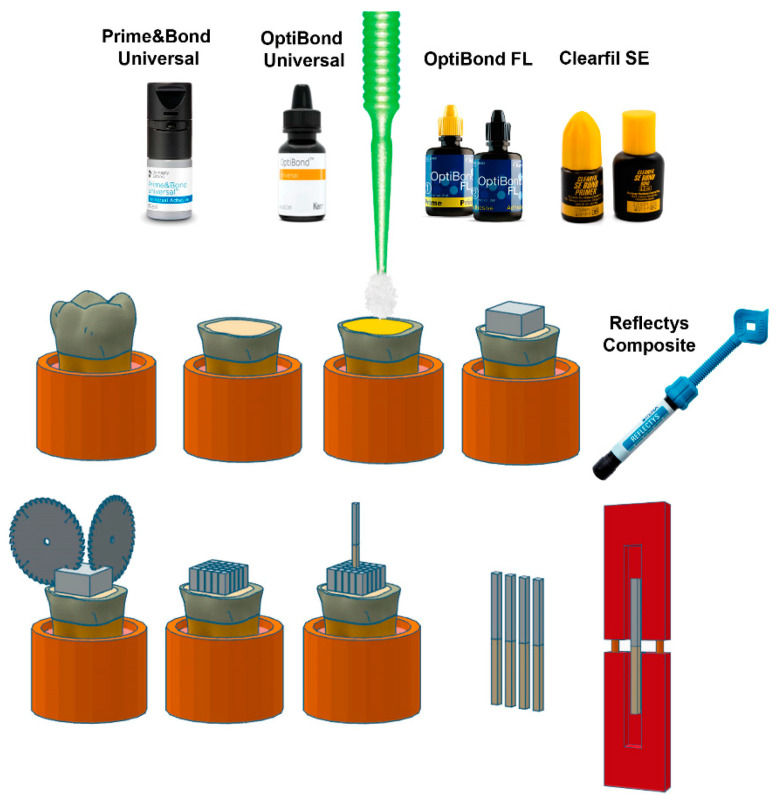
Representation of the tooth preparation, bonding procedure, and resin–dentin beams tested in the current manuscript.

Following adhesion procedure, specimens were stored in distilled water at 37 °C for 24 h. Then, by means of a low-speed precision cutting machine (EXAKT Vertriebs GmbH, Norderstedt, Germany), each tooth was longitudinally segmented across the bonded interfaces in the bucco-lingual and mesio-distal orientations to create resin–dentinal beams with a cross-sectional area of almost 1.0 mm × 1.0 mm (Figure 2).

According to ISO/TS 11405, a determined number of resin–dentin beams (n = 12) from each tooth was assigned to be calculated immediately or following storage in distilled water at 37 °C for a period of 6 months, as revealed in Table S1.

The beams were then secured to a Geraldeli’s jig for μTBS testing by means of cyanoacrylate resin (Zapit Dental Ventures of North America, Corona, CA, USA), and they were put over a tensile force by means of a universal testing machine (YLE GmbH Waldstraße, Bad König, Germany) at a crosshead speed of 1.0 mm/min with a load cell of 500 N until failure [18]. Each failed sample was measured with a digital caliper using a precision of 0.01 mm (Model CD-6BS Mitutoyo, Tokyo, Japan). The failure load of each individual specimen (N) was divided by the mean cross-sectional area (mm^2^), and the results were defined in MPa. The mean bond strength of the examined resin–dentinal beams from each tooth was considered as the value for that tooth.

### 2.4. Failure Mode Analysis

Following the μTBS assessment, the mode of failure for each specimen was examined by means of an optical numeric microscope (Keyence, Osaka, Japan). In order to express the type of fracture, the use of a VHX-5000 software is required for the evaluation of the percentage of each area at 150× magnification. The failures were categorized into adhesive, cohesive (failure in the composite or dentin), and mixed (as one area showed cohesive fracture, though other areas revealed an adhesive failure) failure modes [3].

### 2.5. Scanning Electron Microscopy

After storage period in distilled water for 24 h, three resin–dentin beams were used to analyze the composite–dentin interface morphology of specimens of each group. Afterwards, the interface between resin composite and dentinal structure was etched by means of 37% phosphoric acid for a period of 10 s, and then washed with distilled water for 10 s, and submerged in a 2.5% sodium hypochlorite solution for 3 min [19]. Later, the tested samples were washed with distilled water and dehydrated in a succession of ethanol solutions (25, 50, 75, and 100%). Thereafter, all samples were directly moved to a critical point drying machine (Balzers 030, Shimadzu, Kyoto, Japan) for desiccation. These specimens were subsequently attached on aluminum SEM stubs and sputter-coated with a ration of (20/80) gold–palladium alloys by means of a sputtering device (Hummer JR, Technics, CA, USA). Finally, the prepared specimens were examined using the Quanta 250 FEG SEM (FEI Company, Eindhoven, The Netherlands) functioning at an accelerating voltage (10 kV) of the electrons and at different magnifications.

### 2.6. Statistical Analysis

The Sigma Plot (Version 12, Systat, San Jose, CA, USA) was employed for the statistical analyses. The data experienced analysis to assess the normal distribution and homogeneity of variance. To evaluate the influence of the bonding agent and the application modalities (MTLA with ABT) on the μTBS to dentinal substrate, a two-way analysis of variance (ANOVA) was conducted. The bond strength was separately examined after 24 h and 6 months of storage. Multiple comparisons were performed using Tukey’s post hoc test. A significance level of α = 0.05 was used for all the analyses.

## 3. Results

### 3.1. Micro-Tensile Bond Strength Testing

Table 2 recapitulates the values attained for the μTBS following aging for 24 h, conferring to the material and the technique applied. According to the two-way ANOVA, both factors tested were significant (adhesive, *p* = 0.005; technique, *p* < 0.001, with an interaction between the factors, *p* = 0.005).

For CSE, there were no significant differences in the dentinal bond strength within the different techniques used (*p* > 0.05). For OBFL, OBU, and PBU, statistically significant higher values were perceived for the A technique (*p* < 0.05) when compared to the other techniques. Plus, there were no significant differences between the APP, AAP, and AAA techniques (*p* > 0.05) for OBFL and PBU. For OBU, there were no significant differences between the A and AAA techniques (*p* > 0.05).

Regarding the effect of the adhesive system within each technique, for the A and the AAP techniques, there were no significant variances between the different adhesive systems tested (*p* > 0.05). PBU had statistically lower values in comparison to the other adhesive systems when used with the APP and AAA techniques (*p* < 0.05).

Table 3 shows the values acquired for the μTBS to dentin following a period of 6 months of aging according to the material and the technique applied in this study. The results from the two-way ANOVA showed that the factor adhesive was not significant (*p* = 0.282); on the other hand, the factor technique and the interaction between the factors were significant (*p* < 0.001 and *p* = 0.005, respectively).

For CSE, OBU, and PBU, the A technique showed significant higher values than the other techniques (*p* < 0.01). For the same adhesives, the differences in the bond strength values for AAA, AAP, and APP were not significant (*p* > 0.05). For OBFL, the highest values were observed for the A and the AAA techniques, which were statistically higher than AAP and APP (*p* < 0.001).

Tukey’s post hoc test displayed significant differences within the AAA technique, where OBFL achieved statistically higher values than the other adhesives (*p* < 0.001). On the other hand, for the rest of the techniques tested, there were no significant differences between the bond strength values (*p* > 0.05).

Table 4 shows the comparison of the bond strength values following 24 h and 6 months of aging for each adhesive system and technique tested. CSE showed bond strength stability only when applied with the A technique (*p* = 0.537). For OBFL, no significant changes in the bond strength were observed for APP and AAA (*p* > 0.05). For OBU, this behavior was observed only in the AAP technique (*p* = 0.08). Finally, for PBU, all the techniques used resulted in bond strength stability over time (*p* > 0.05).

### 3.2. Failure Mode Analysis

The numbers of fracture modes are shown in Table 5, Table 6, Table 7 and Table 8.

The failures were mainly adhesive or mixed fractures in all of the bonding systems evaluated. Higher mixed failures were reported in the specimens with higher bond strengths when compared to higher adhesive failure in the samples with lower bond strengths across all adhesive systems and MTLA specimens.

### 3.3. Scanning Electron Microscopy of Resin–Dentin Interface

**Figure 3:**
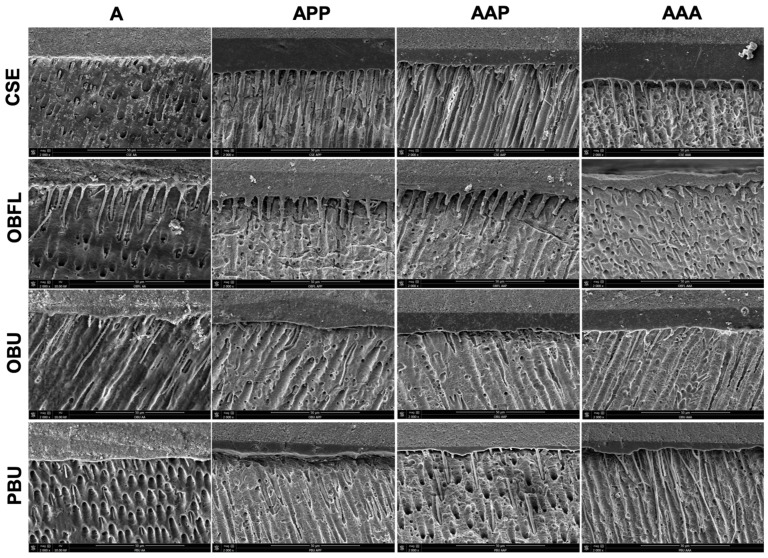
Scanning electron microscopy images (×2000 magnification) determine the adhesive layer and resin tag infiltration of the distinctive dental adhesive systems examined with the modified triple-layer application and the control group. Clearfil SE Bond (CSE); OptiBond FL (OBFL); OptiBond Universal (OBU); Prime&Bond Universal (PBU); Single active application (A); triple application, Active–Passive–Passive (APP); triple application, Active–Active–Passive (AAP); triple application, Active–Active–Active (AAA).

Representative SEM micrographs of the dentinal–resin interface of the different adhesive systems tested are presented in Figure 3. Differences in the thickness of the adhesive layer and resin tags (RT) formation can be observed among the different techniques tested. Thicker adhesive layers are observed in the APP, AAP, and AAA groups in comparison with the A group. A higher number of RT is observed in the AAA groups. Only the OBFL adhesive showed the formation of RT in all of the modalities tested.

## 4. Discussion

Adhesive dentistry became a trend decades ago, and with this rise in popularity, multiple techniques were developed in an attempt to reduce the sensitivity of adhesives [12]. In particular, MLA was proposed to enhance the monomer infiltration of adhesives to dentin [13]. Yet, the variation of the application of each layer, either in an active or a passive manner, was not previously assessed in the literature for triple-layer applications. The result of the present investigation led to the partial rejection of the null hypothesis since the dentinal bonding properties of some contemporary adhesives tested were affected by the effect of MTLA.

Among all tested adhesives, at 24 h, only CSE did not display any significant variances in the bond strength within the different techniques used (*p* > 0.05). CSE is an SE adhesive, which has a high concentration of acidic monomers inside an aqueous solution. These monomers release H+ ions and etch the dental substrate in synchronization with their diffusion into the substrate [20]. Since a variation in the bond strength was not observed, the claim that CSE can withstand one coat of application as well as MTLA can be made.

Starting with the second layer, additional benefits for the bond strength will be negligible, and the outcome will resemble that of the one-layer application. To clarify, when a second coat of adhesive was applied to the dentin, a large number of H+ ions were liberated to permit the etching agent to operate for a longer duration [21]. However, previous reports [22,23] found that an increased SE system action time through a surge in H+ ions did not develop an observable effect on the bond strength, which supports the outcomes of this study. Thus, no difference was discovered between the tested groups (A, APP, AAP, and AAA), although there was a slight decline in the bond strength only when the passive application (PA) was incorporated between layers (APP and AAP). So, in theory, the amount of H+ ions does not have an effect on the immediate bond strength of the CSE bond regardless of the number of layers applied.

For OBFL, OBU, and PBU, the values observed for the A technique were higher in a significant manner (*p* < 0.05). On the other hand, there were no significant differences between the APP, AAP, and AAA techniques (*p* > 0.05) for OBFL and PBU. For OBU, there were not any statistically differences between the A and AAA techniques (*p* > 0.05). With that said, one coat of each of these adhesives (OBFL, OBU, and PBU) is enough to effectively produce an HL enclosed by an adhesive coat that will offer appropriate pairing to successively applied resin composite, although a drop in the dentin bond strength can occur when two or more coatings are used. Also, increasing the number of layers could result in deficiencies in the application technique; for example, the clinician might not provide sufficient drying time for the primers or the adhesives. Further, hydrophilic components inside the adhesives may accumulate between layers and lead to adhesive degradation [16].

Regarding the effect of the adhesive system within each technique, there were no significant differences amongst the different adhesive systems (*p* > 0.05) for the A and AAP techniques. PBU had statistically significant lower values in comparison with the other adhesive systems when used with the APP and AAA techniques (*p* < 0.05). All things considered, the PBU application was affected when the second layer and third layer were similar, for example, when AA is used twice after the first ABT, with the same being said for true passive applications in a row at the second and third layer. With APP, the bond strength was at its lowest values. This could be clearly related to the adhesive itself, which might not be able to withstand MTLA. UAs raise the capability for demineralization with the substrates by taking up their water content, and whenever the water level increases in the adhesive, the polymerization of the adhesive coat applied to the substrate becomes less sufficient. This means that each time a layer of PBU is added, the amount of water surges [3]. Plus, in this study, photopolymerization was performed only at the end of the MTLA, which can be attributed to the high sensitivity of PBU to these techniques after 24 h. The recommended application time for PBU is 20 s with a slight agitation [3]. This improves monomer infiltration into the dentinal substrate as well as solvent evaporation. This could justify the reduced benefit of further layers (MTLA in this case). Another factor for the results of PBU is an insufficient air-drying time, which is recommended to be around 15 s to 30 s for some UAs. Strong air drying enhances the mechanical characteristics and solvent evaporation, which leads to a stronger adhesive layer at the dentinal interface [13]. Clinicians are recommended to eliminate any remaining solvent to reach the optimal bond performance of the cured adhesive, as well as to carefully choose the material and solvent types. Seemingly, for PBU, the clinician should apply only one coat in an active mode.

After 6 months, for CSE, OBU, and PBU, the A technique showed statistically higher values than the other techniques (*p* < 0.01). For the same adhesives, the differences in the bond strength values for AAA, AAP, and APP were not significant (*p* > 0.05). These adhesive systems were applied in SE mode in this study, thus explaining that this strategy did not support the MTLA, which has lower values when compared to a single coat application. SE adhesive systems contain water or hydrophilic resin to enhance monomer penetration into the dentin [24,25], but this hydrophilicity can induce water sorption and an eventual instability of the HL with time [26]. For CSE, the MTLA may not have added benefits on the dentinal bond strength due to this adhesive having a thicker adhesive layer when applied in only one coat. The relatively thick adhesive layer could scatter the stress distribution at the interface among dentin and resin composites during testing, successively raising the bond strength of the adhesive and justifying the choice of not adding a second layer [27,28].

Further, the UAs tested in this research were applied in an SE mode, and if some solvents remain after evaporation (OBU = 30–60% acetone, ~5–10% ethanol; PBU = 5–24.5% water, 10–24.5% isopropanol) [16,29], the hydrolysis of resin polymers and the enzymatic degradation of collagen may occur over time [3,30]. The accumulation of solvents between layers in the MTLA is detrimental to these UAs. Another explanation might be that the thick smear layer prevents the active functional monomers from penetrating and interacting with the dentin to create a suitable HL [31]. Accordingly, the poorly dissolved smear layer weakens the adhesive layer. All in all, the bonding agents used in the SE mode in this manuscript (CSE, OBU, and PBU) benefits from a one-layer application after 6 months of water storage.

For OBFL, the highest values were observed for the A and AAA techniques after aging (6 months), which were statistically higher than AAP and APP (*p* < 0.001). OBFL is an E&R adhesive with an ability to strongly bond to dentin over long storage times [32]. A previous study stated that the bond strength was improved through E&R adhesives with MLA [33]. This was in agreement with the findings of this study only with AAA for MTLA. The solvent content inside the adhesive and the comonomer infiltration into dentin are the key players in enhancing the bond strength if applied properly to the dentin. Plus, incomplete evaporation of the water and ethanol solvents in OBFL can significantly reduce the bond strength. This can explain why A and AAA resulted in superior bond strength values compared to the other techniques due to sufficient solvent removal [34]. In addition, this is due to the inclusion of filler in the composition of OBFL and the close attention to the application technique [3,35]. This high filler load inside OBFL strengthens the HL and may serve as a shock absorber [36], which might also clarify its high dentin bond strengths for both the A and AAA techniques. For OBFL specifically, the manufacturer quotes the following for the primer component: “apply with a light brushing motion for 15 s” [3]. Based on what was established, the inclusion of PA even in one layer of MTLA (APP and AAP) led to a drop in the bond strength.

The factor adhesive was only significant for the AAA technique, where OBFL achieved statistically higher values than the other adhesives (*p* < 0.001) following 6 months of distilled water storage. On the other hand, for the rest of the techniques tested, there were no significant differences between the bond strength values (*p* > 0.05). In the general run of things, OBFL was the only adhesive affected by one of the techniques tested in this research. Specifically, the AAA favored OBFL. This is attributable to the fact that E&R adhesives offer superior bond strengths when compared to SE following multiple adhesive coats [37]. A prior examination by Hashimoto et al. noted that four succeeding layers of E&R adhesives without the polymerization of each layer strengthen the bond strength [38]. This was in agreement with the discovery of this research when AAA was used. 

After 6 months, all adhesives were unfavorably influenced by a PA. This can be related to the enhanced bond strength and the value of the adhesion, which is only attainable by the continuous rubbing of OBFL. The agitation of an adhesive maintains the fresh acidic solution in conjunction with the dental substrate, leads to a high rate of monomer infiltration, and promotes solvent evaporation, thereby reinforcing the adhesion to dentin [3,12,39,40]. This perhaps explains the higher adhesion with the A technique for some adhesives. For that reason, no difference was found for the tested adhesive (OBFL) with this technique. Further, repeating the A technique by means of three layers (AAA) could accumulate the benefits of the agitation application only for OBFL. Hardan et al. claimed that E&R adhesives were improved by the MLA technique immediately and after long-term storage [12]. Adding to these clarifications, the presence of fillers in OBFL [3,36] with three layers of applications (AAA) might be the reason for this acceptable bond strength. High filler loads will affect bonding, as they might form clusters and hinder infiltration within the collagen matrix [41]. However, the combination of active agitation with MLA was a good choice for the highly filled OBFL. This explains that this specific technique will perform as a layer reinforced (LR)-MTLA.

So, the combination of E&R adhesive, the AAA with continuous rubbing, and the presence of fillers inside the adhesive (OBFL), ameliorates the long-term bond performance to dentin when choosing the MTLA as the application modality. 

For the comparison of the bond strength values at 24 h and 6 months of aging, CSE showed bond strength stability only when applied with the A technique (*p* = 0.537). A decline in the bond strength was observed for all of the tested MTLAs. This can be interpreted by the fact that this adhesive system is based on 10-MDP and 2-hydroxyethyl methacrylate (HEMA) [3]. In the case of using the MTLA, the functional monomers inside CSE, which are considered as adhesion promoters, will affect the stability of the bond with time. The hydrophilic characteristic of these monomers, like HEMA, raises the dentinal bond strength of the adhesives, and some functional monomers such as 10-MDP could bond chemically to calcium [42], while as advocated in the scientific literature, 10-MDP-containing SE adhesives have better bond durability [43]. This was only observed in the current study when the A technique was used. Hence, 10-MDP alone was not the reason for the stability or the decline of the bond strength. It appears that the impact of HEMA on bond weakening and water sorption is tremendous in a way that negates the positive influence of 10-MDP on the dental substrate when used with MTLA. This confirms the benefit of one-layer application by means of agitation for CSE (A technique), and shows the variation of bond stability between the tested techniques in this study. When multiplying the applied layers, the activity and the concentration of these monomers increases and negatively affects the outcome of the aged bond strength. Generally speaking, and according to previous investigation, nanolayering could be affected by agitation, 10-MDP concentration, and the construction of the functional monomers presented in the adhesive [3,7,13].

For OBFL, when comparing the value between 24 h and 6 months, no significant changes in the bond strength were observed for APP and AAA (*p* > 0.05). Regarding APP, the bond strength was immediately lower and maintained its low value over time. However, for AAA, the bond strength was preserved, saving the higher long-term bond performance among all of the tested MTLAs with OBFL. This sheds light on a novel technique for the OBFL with long-term stability. This technique combines the advantages of both MLA and ABT and is effective for this specific bonding. Suitably, it can be called the LR-MTLA. Referring to a previous report [44], bonding agents presenting dental etchants, primers, and hydrophobic adhesives in individual bottles with no solvent content in the adhesives are better in respect to adhesion stability, as long as the effect of the hydrophilic mixtures on the level of polymer conversion is absent, with OBFL being an example of this [45]. This could justify why the bond strength of OBFL did not decrease between 24 h and 6 months in APP and AAA. The decrease in the bond strength for the A and AAP techniques affirmed that OBFL supports MLA [12], but the condition should be the agitation of all of the layers applied. Additionally, the deterioration of the dentin bond strength of OBFL is linked to different monomers presented in the formulation of primers like GPDM and HEMA that can influence the features of the polymers obtained, the bonding potential, and the degradation over time [46,47].

For OBU, the stability of the bond strength was observed only for the AAP technique (*p* = 0.08), which already had low values after 24 h and after 6 months as well. This could be linked to the functional monomer, GPDM, which seems to be sensitive to the technique used in this study. Although GPDM was reported to be adhered to HAp, it was unable to form a monomer-calcium salt that can remain stable with time [48]. Likewise, the bond between GPDM and HAp was shown to be weak, and this was documented in several articles [48,49,50]. HEMA, which is found in OBU, is compatible with the dentinal structure, and its hydrophilicity contributes to the ease of its infiltration into the demineralized substrate, but also makes it vulnerable to water sorption and hydrolysis [51]. Moreover, OBU contains acetone, which can reduce the bond strength if not sufficiently evaporated [3]. This can explain the low bond strength values obtained after 6 months.

Finally, for PBU, all of the techniques used resulted in bond strength stability over time (*p* > 0.05). So, this adhesive can only support one coat (A), since MTLA already had a low bond strength and stayed that way after 6 months. The stable bond strength was linked to dipentaerythritol pentaacrylate phosphate (PENTA) [52,53], 10-MDP [54], and methacrylamide [55] monomers inside the composition of this specific bonding. PENTA was proven to be more stable than MDP, where the bond strength was maintained at the end of the adhesive’s shelf life [53]. It could be theorized that, contrary to 10-MDP monomers, the existence of five vinyl groups inside the chemical structure of PENTA might enhance its resistance to water degradation. Therefore, four vinyl groups will still be present for connection maintenance with the phosphate group following hydrolysis, which enables copolymerization and adhesion to the dentin simultaneously [52,56]. In this respect, PBU maintained its stability. PBU is an adhesive that does not contain HEMA in its formulation, and this particular composition can aid in effectively eliminating water during the air-drying process [3,54]. Additionally, methacrylamide monomers were produced to substitute HEMA in PBU in order to avoid phase separation and reduce water sorption inside the adhesive [30,55].

Following the μTBS analysis, the failure mode was assessed in all of the groups tested in this study. Mostly, the adhesive failures were predominantly adhesive or mixed in all the bonding systems evaluated. The bond strength test used a load force qualified to pass through the dentin and the resin composite before reaching the adhesive interface, with resulting stress intensity at these locations [57], initiating a relatively higher ratio of mixed failures. Additionally, this declaration may also designate a suitable dentinal hybridization [58]. After aging in distilled water for a period of 6 months, the failure analysis was commonly adhesive. This is related to the aging of an adhesive layer, developing further adhesive fracture compared to the baseline mode of failure (after 24 h) across all adhesive systems and MTLA specimens [59].

The SEM observations denoted, after 24 h, an elevated number of RT amongst the AAA groups compared to the other techniques where passive applications were applied. When passing from A to AAA, the resin penetration increased, except for OBFL, where the infiltration was higher in all the techniques tested. This could be due to the fact that ABT allows for a superior monomer penetration inside the branches of dentinal tubules [3,12]. Further, this was perhaps because of the removal of the non-penetrated resin plugs inside the tags via the acids and bases used to dissolve the dentin from the resin [60]. Moreover, this might be linked to the statement that the ABT has been proven to increase the interaction among adhesive systems and dentinal substrates, varying the biochemical characteristics of dentin in a positive way, and simplifying the infiltration of the material inside the inter and peritubular region [3,12]. The OBFL adhesive exhibited a unique characteristic in the tests—it consistently generated RT across all the modalities examined. This behavior can be attributed to the specific etching step that this adhesive necessitates for the bonding process [44]. Previous data supports the notion that this etching step plays a critical role in promoting a deeper penetration of the adhesive into the dentin substrate [61]. This deeper penetration results in longer RT and the formation of thicker HL [62]. Considering this, regardless of the application modality employed, the use of phosphoric acid for removing the smear layer and smear plugs led to enhanced adhesive infiltration. This step also facilitated the adhesive’s penetration into the dentin tubules, thereby improving both the length and morphology of the RT [63].

While the adhesive layer’s thickness could be perceived as a possible factor influencing the bond strength, particularly in terms of enhancing stress distribution within the body assembly, there remains an ongoing debate regarding the correlation between the thickness of the adhesive layer and the bond strength [64]. It was previously stated that the increase in the adhesive layer thickness would efficiently distribute stress at the interface between the composite material and the tooth [11]. Despite this, differences in the composition (solvent agents and fillers) can lead to differences in the results expected, as observed in the present study. Considering this, the exact application of an adhesive system is material-dependent.

Some limitations could be drawn from this research. First of all, only a reduced number of adhesive systems were tested, and it is worth mentioning that a representative from the two-step E&R adhesive system is missing. Also, more UAs should be included in future works. In addition to these, a variation in the methodology, in terms of the photopolymerization of each layer, should be tested in the future. Further studies might be accomplished with an accurate, safe, and non-destructive method like micro-computed tomography (micro-CT) instead of SEM. The assessments of the tag density, length, and size, in addition to the thickness of the HL, might be affected by the location of the dentin tubules. Tubule diameters and densities rise from the dentin/enamel junction to the central dentinal area. So, all of the SEM interpretations might be interrelated to the examined anatomical region. Assessments like cytotoxicity tests must be explored in future studies. Bond strength analyses with more aging procedures could be conducted, such as thermocycling, in an attempt to look for extra signs of adhesive degradation at the interface. It should be highlighted that the main cause for the failure of dental restorations is nanoleakage formed by a reduced dentin bond strength. Therefore, additional research should be performed to validate the present preliminary outcomes.

## 5. Conclusions

Based on the findings of this study, the subsequent conclusions can be addressed:

1. The laboratory adhesive properties were mostly material-dependent.
2. For better bond strength performance, CSE, OBU, and PBU were benefited by the A technique; for OBFL, the use of the AAA technique could be recommended to achieve stability in the adhesive layer.
3. The combination of ABT with an MLA was a good choice for the highly filled OBFL. This explains that the LR-MTLA was considered a novel approach in the field of adhesive dentistry with an acceptable bond stability after 6 months.
4. Clinicians must consider the chemistry and the physical features of each adhesive system in an attempt to determine its ideal performance before applying the MTLA.

## Figures and Tables

**Table 1:** Manufacturers and compositions of the adhesives used.

Material	Classification	Composition *	Manufacturer	Recommendation by the Manufacturer for Adhesive Application
PBU	Mild Universal pH = 2.5	10-MDP, PENTA, isopropanol, water, photoinitiator, bi- and multifunctional acrylate	Dentsply DeTrey GmbH, Konstanz, Germany	Apply PBU to all cavity surfaces. Avoid pooling. Keep PBU slightly agitated for 20 s. Evaporate solvent with air for at least 5 s. Light cure.
OBU	Universal pH = 2.5–3.0	Acetone, HEMA, GDMA, ethanol, GPDM	Kerr Co, Orange, CA, USA	Using the disposable applicator brush, apply a generous amount of OBU adhesive to the enamel/dentin surface. Scrub the surface with a brushing motion for 20 s. Dry the adhesive with gentle air first and then medium air for at least 5 s with oil-free air. The surface should have a glossy uniform appearance. If not, repeat the bonding and drying steps. Light cure.
OBFL	Three-step etch-and- rinse pH primer: 1.9; pH bonding: 6.9	Etchant: 37.5% H_3_PO_4_ Primer: HEMA, GPDM, MMEP, water, ethanol, CQ, and BHT Adhesive: Bis-GMA, HEMA, GDMA, CQ, and filler (fumed SiO_2_, barium aluminoborosilicat, Na_2_SiF_6_), coupling factor A174	Kerr Co, Orange, CA, USA	Apply OBFL primer using an applicator brush over enamel and dentin surfaces with a light scrubbing motion for 15 s. Gently air dry for approximately 5 s. At this point, the dentin surface should have a slightly shiny appearance. Using a new applicator brush, apply OBFL adhesive to the prepared enamel and dentin surfaces with a light scrubbing motion for 15 s, creating a thin coating. Gently air dry for approximately 5 s. Light cure.
CSE	Two-step self-etch pH primer = 1.76 pH bond = 2	Primer: 10-MDP, HEMA, hydrophilic dimethacrylate, CQ, DEPT, water, ethanol Bond: MDP, HEMA, Bis-GMA, hydrophobic dimethacrylate, CQ, DEPT, silanized colloidal silica	Kuraray Noritake Dental Inc., Tokyo, Japan	Apply primer for 20 s. Dry with mild air flow. Apply bond. Apply air flow gently. Light cure.

* Based on companies’ MSDS. 10-MDP = 10-methacryloyloxydecyl dihydrogen phosphate; PENTA = dipentaerythritol pentaacrylate phosphate; HEMA = 2-hydroxy ethyl methacrylate; GDMA = glycerol-dimethacrylate; GPDM = glycero-phosphate dimethacrylate; MMEP = methacryloyloxy-ethyl-dihydrogen phosphate; CQ = camphorquinone; BHT = butyl hydroxy toluene; Bis-GMA = bisphenol A-glycidyl methacrylate; DEPT = N,N-diethyl-p-toluidine; Clearfil SE Bond (CSE); OptiBond FL (OBFL); OptiBond Universal (OBU); Prime&Bond Universal (PBU).

**Table 2:** Mean and standard deviation of the micro-tensile bond strength test to dentin of the different application modalities for the dental adhesive systems at 24 h of aging.

Technique	CSE	OBFL	OBU	PBU
A	^X^19.02 (3.19) ^a^	^X^29.66 (5.25) ^a^	^X^28.3 (5.02) ^a^	^X^26.16 (8.9) ^a^
APP	^X^16.86 (2.74) ^a^	^XY^11.87 (4.66) ^b^	^XY^13.6 (2.25) ^b^	^Y^7.34 (2.2) ^b^
AAP	^X^17.12 (5.20) ^a^	^X^15.25 (2.79) ^b^	^X^12.5 (3.73) ^b^	^X^10.17 (3.1) ^b^
AAA	^X^19.77 (2.98) ^a^	^XY^17.16 (6.39) ^b^	^X^20.6 (8.73) ^ab^	^Y^11.49 (2.8) ^b^

Different uppercase letters indicate the presence of statistically significant differences for each row (*p* < 0.05). Different lowercase letters indicate the presence of statistically significant differences for each column (*p* < 0.05). Clearfil SE Bond (CSE); OptiBond FL (OBFL); OptiBond Universal (OBU); Prime&Bond Universal (PBU); Single active application (A); triple application, Active–Passive–Passive (APP); triple application, Active–Active–Passive (AAP); triple application, Active–Active–Active (AAA).

**Table 3:** Mean and standard deviation of the micro-tensile bond strength test to dentin of the different application modalities for the dental adhesive systems at 6 months of aging.

Technique	CSE	OBFL	OBU	PBU
A	^X^17.60 (3.75) ^a^	^X^13.73 (3.12) ^a^	^X^16.71 (6.13) ^a^	^X^20.11 (2.95) ^a^
APP	^X^7 (3.98) ^b^	^X^10.26 (5.8) ^b^	^X^6.75 (3.26) ^b^	^X^7.51 (3.75) ^b^
AAP	^X^10.31 (4.22) ^b^	^X^8.38 (1.47) ^b^	^X^8.57 (2.46) ^b^	^X^6.7 (2.74) ^b^
AAA	^Y^10.43 (2.77) ^b^	^X^18.03 (5.26) ^a^	^Y^9.24 (1.76) ^b^	^Y^9.06 (4.24) ^b^

Different uppercase letters indicate the presence of statistically significant differences for each row (*p* < 0.05). Different lowercase letters indicate the presence of statistically significant differences for each column (*p* < 0.05). Clearfil SE Bond (CSE); OptiBond FL (OBFL); OptiBond Universal (OBU); Prime&Bond Universal (PBU); Single active application (A); triple application, Active–Passive–Passive (APP); triple application, Active–Active–Passive (AAP); triple application, Active–Active–Active (AAA).

**Table 4:** Comparison of the mean and standard deviation of the micro-tensile bond strength test to dentin of the different application modalities for the dental adhesive systems examined at 24 h and 6 months of aging.

Technique/Adhesive	Aging	
CSE	24 h	6 months
A	19.02 (3.19) ^X^	17.60 (3.75) ^X^
APP	16.86 (2.74) ^X^	7 (3.98) ^Y^
AAP	17.12 (5.20) ^X^	10.31 (4.22) ^Y^
AAA	19.77 (2.98) ^X^	10.43 (2.77) ^Y^
OBFL	24 h	6 months
A	29.66 (5.25) ^X^	13.73 (3.12) ^Y^
APP	11.87 (4.66) ^X^	10.26 (5.8) ^X^
AAP	15.25 (2.79) ^X^	8.38 (1.47) ^Y^
AAA	17.16 (6.39) ^X^	18.03 (5.26) ^X^
OBU	24 h	6 months
A	28.3 (5.02) ^X^	16.71 (6.13) ^Y^
APP	13.6 (2.25) ^X^	6.75 (3.26) ^Y^
AAP	12.5 (3.73) ^X^	8.57 (2.46) ^X^
AAA	20.6 (8.73) ^X^	9.24 (1.76) ^Y^
PBU	24 h	6 months
A	26.16 (8.9) ^X^	20.11 (2.95) ^X^
APP	7.34 (2.2) ^X^	7.51 (3.75) ^X^
AAP	10.17 (3.1) ^X^	6.7 (2.74) ^X^
AAA	11.49 (2.8) ^X^	9.06 (4.24) ^X^

Different uppercase letters indicate the presence of statistically significant differences between 24 h and 6 months. Clearfil SE Bond (CSE); OptiBond FL (OBFL); OptiBond Universal (OBU); Prime&Bond Universal (PBU); Single active application (A); triple application, Active–Passive–Passive (APP); triple application, Active–Active–Passive (AAP); triple application, Active–Active–Active (AAA).

**Table 5:** Failure mode analysis of the Clearfil SE Bond adhesive system tested following the bond strength test.

Technique	Material	Aging	Failure Types			
			Adhesive	Mixed	Cohesive Resin	Cohesive Dentin
A	CSE	24 h	14	16	0	0
		6 months	13	15	1	1
APP	CSE	24 h	10	14	3	3
		6 months	17	9	2	2
AAP	CSE	24 h	13	16	1	0
		6 months	17	11	2	0
AAA	CSE	24 h	11	15	2	2
		6 months	16	10	2	2

Clearfil SE Bond (CSE); Single active application (A); triple application, Active–Passive–Passive (APP); triple application, Active–Active–Passive (AAP); triple application, Active–Active–Active (AAA).

**Table 6:** Failure mode analysis of the OptiBond FL adhesive system tested following the bond strength test.

**Technique**	**Material**	**Aging**	**Failure Types**			
			**Adhesive**	**Mixed**	**Cohesive Resin**	**Cohesive Dentin**
A	OBFL	24 h	11	19	0	0
		6 months	16	12	1	1
APP	OBFL	24 h	15	15	0	0
		6 months	16	14	0	0
AAP	OBFL	24 h	13	15	2	0
		6 months	18	12	0	0
AAA	OBFL	24 h	11	16	2	1
		6 months	14	16	0	0

OptiBond FL (OBFL); Single active application (A); triple application, Active–Passive–Passive (APP); triple application, Active–Active–Passive (AAP); triple application, Active–Active–Active (AAA).

**Table 7:** Failure mode analysis of the OptiBond Universal adhesive system tested following the bond strength test.

Technique	Material	Aging	Failure Types			
			Adhesive	Mixed	Cohesive Resin	Cohesive Dentin
A	OBU	24 h	11	17	1	1
		6 months	18	12	0	0
APP	OBU	24 h	14	16	0	0
		6 months	20	10	0	0
AAP	OBU	24 h	15	15	0	0
		6 months	21	9	0	0
AAA	OBU	24 h	11	13	3	3
		6 months	18	12	0	0

OptiBond Universal (OBU); Single active application (A); triple application, Active–Passive–Passive (APP); triple application, Active–Active–Passive (AAP); triple application, Active–Active–Active (AAA).

**Table 8:** Failure mode analysis of the Prime&Bond Universal adhesive system tested following the bond strength test.

Technique	Material	Aging	Failure Types			
			Adhesive	Mixed	Cohesive Resin	Cohesive Dentin
A	PBU	24 h	10	20	0	0
		6 months	12	18	0	0
APP	PBU	24 h	15	15	0	0
		6 months	14	15	0	1
AAP	PBU	24 h	14	16	0	0
		6 months	20	9	1	0
AAA	PBU	24 h	14	16	0	0
		6 months	15	14	1	0

Prime&Bond Universal (PBU); Single active application (A); triple application, Active–Passive–Passive (APP); triple application, Active–Active–Passive (AAP); triple application, Active–Active–Active (AAA).

## Data Availability

The data presented in this study are available upon reasonable request from the author (R.B.).

## Supplementary Materials

The following supporting information can be downloaded at https://www.mdpi.com/article/10.3390/jfb14100522/s1, Table S1: Micro-tensile bond strength test groups.
