# Supplementary material for: Effect of Modified Triple-Layer Application on the Bond Strength of Different Dental Adhesive Systems to Dentin
Source: J Funct Biomater. 2023 Oct 17;14(10):522. doi: 10.3390/jfb14100522 (PMC10607670; doi:10.3390/jfb14100522)
Supplement: Supplementary file 1 [file jfb-14-00522-s001.zip › jfb-2649761-supplementary.pdf]

Table S1. Micro-tensile bond strength test groups.

Group	Adhesive System	Application Technique	Teeth (*n*)	Slabs/24h	Slabs/6 months
1	CSE	A	5	30	30
2	CSE	APP	5	30	30
3	CSE	AAP	5	30	30
4	CSE	AAA	5	30	30
5	OBFL	A	5	30	30
6	OBFL	APP	5	30	30
7	OBFL	AAP	5	30	30
8	OBFL	AAA	5	30	30
9	OBU	A	5	30	30
10	OBU	APP	5	30	30
11	OBU	AAP	5	30	30
12	OBU	AAA	5	30	30
13	PBU	A	5	30	30
14	PBU	APP	5	30	30
15	PBU	AAP	5	30	30
16	PBU	AAA	5	30	30
		Total:	80	480	480

Clearfil SE Bond (CSE); OptiBond FL (OBFL); OptiBond Universal (OBU); Prime&Bond Universal (PBU); Single active application (A); triple application, Active–Passive–Passive (APP); triple application, Active–Active–Passive (AAP); triple application, Active–Active–Active (AAA).
